# Evaluation of the Normal Measurements of Orbital Structures in Healthy Adult Individuals by Using a Magnetic Resonance Imaging Method

**DOI:** 10.3390/tomography10110125

**Published:** 2024-10-22

**Authors:** Nazire Kiliç Şafak, Sibel Tepecik

**Affiliations:** 1 Department of Anatomy, Faculty of Medicine, Çukurova University, 01010 Adana, Turkey; 2 Department of Radiology, Yuregir State Hospital, 01010 Adana, Turkey; radiologistsibeltepecik@gmail.com

**Keywords:** extraocular muscles, magnetic resonance imaging, orbit

## Abstract

Background: This study aims to determine the normal values of orbital structures according to sex in healthy adults. Methods: Diameters of extraocular muscles, the width of the optic nerve sheath diameter, the length of the inter-zygomatic line, ocular bulb length, and globe position were measured in the T1-weighed MR (magnetic resonance) images in 204 orbits of 102 individuals. Results: The mean values of the diameters of the extraocular muscles in males and females were as follows: medial rectus, 3.96 ± 0.52 and 3.58 ± 0.53 mm; lateral rectus, 3.47 ± 0.61 and 3.15 ± 0.48 mm; inferior rectus, 4.47 ± 0.53 and 4.07 ± 0.48 mm; superior rectus, 4.44 ± 0.64 and 4.01 ± 0.56 mm; and superior oblique, 3.68 ± 0.49 and 3.45 ± 0.44 mm. The length of the interzygomatic line in males and females were 102.68 ± 3.89 and 96.95 ± 3.4 mm, the ocular bulb length was 23.33 ± 1.32 and 22.83 ± 1.1 mm, the globe position was 7.66 ± 1.33 and 7.3 ± 1.39 mm, and the width of the optic nerve sheath diameter was 4.65 ± 0.62 and 4.28 ± 0.51 mm, respectively. All measurements were significantly greater for males than for females (*p* < 0.05). Conclusions: We believe that a practical and quantitative method will be provided by this study for the diagnosis and determination of the normative values of orbital structures.

## 1. Introduction

The orbit is a pair of conical-shaped bony cavities bounded by the anterior and middle skull base and the viscerocranium [1]. The orbit surrounds the eyeball. It contains important structures such as muscle, vascular structures, fatty tissue, interstitial tissue, and the optic nerve [2]. The extraocular muscles and optic nerve are important parts of the visual system that transmit visual data between the eye–brain and provide synchronized movements of the eyeball and eyelids [3,4]. Morphometric characteristics of orbital structures may vary according to age, gender, and ethnicity [5]. Determination of the normative values of orbital structures such as extraocular muscles, optic nerve sheath complex, and globe position, particularly through advanced methods such as magnetic resonance imaging, which is advantageous compared to computed tomography (CT), will provide important quantitative data to be used for diagnostic purposes [6,7]. In the literature, studies determining normative values of orbital structures are usually based on western populations [8]. Although there are many studies in the literature addressing various aspects of the extraocular muscle, optic nerve sheath complex, and globe position, there is still a need for comprehensive normative data from the Anatolian population to guide diagnoses and surgical interventions [2,3,4,6,7]. Additionally, related anthropometric measurements are found in the literature. However, these measurements are usually external superficial measurements. In addition to these, there is a need for measurements related to the soft tissue in the internal structure of the orbit and the surrounding bony structure [9].

The retrobulbar space of the orbit contains six extraocular muscles: superior, inferior, medial, and lateral recti, and the superior and inferior oblique muscles. These muscles are responsible for synchronized movements of the eyeball and eyelids [3]. Extraocular muscles are an important reference point for surgical operations of strabismus. It is important to know the morphometric characteristics of the orbital region for the success of this surgery [10]. Thyroid disease is a multifactorial disease affecting orbital structures such as adipose tissue and extraocular muscle with inflammatory changes that can cause disfiguring and sight-threatening injuries [11]. Thyroid disease causes conditions such as exophthalmos, eyelid retraction, lateral flare, strabismus, and optic neuropathy. And all of these conditions are thought to derive from enlargement of the orbital structures [12]. Various inflammatory conditions may occur in orbital structures under neoplastic and vascular conditions, along with enlargement of the extraocular muscles, optic nerve sheath, and superior ophthalmic vein [5]. In the diagnosis of orbital diseases and the identification of pathological changes, it is essential to know the normal size of the extraocular muscles [13].

The sheath surrounding the optic nerve is a continuation of the subarachnoid space, so any change in intracranial pressure can change its diameter. For this reason, the optic nerve sheath diameter is used for diagnosing intracranial pressure [14]. Raised intracranial pressure is closely related to conditions such as stroke, liver failure, meningitis, meningoencephalitis, and brain herniation. Determination of normative values of optic nerve sheath diameter is important for the management of various conditions like these [15,16].

In the evaluation of orbital diseases, radiologic imaging is routinely performed for diagnosis [17]. Ultrasonography measurements have been used in the past to detect diseases affecting the extraocular muscles, but have been shown to be unreliable in the literature. Computed tomography (CT) and magnetic resonance imaging (MRI) are the main techniques used to detect diseases affecting the extraocular muscles [18]. MRI is a non-invasive cross-sectional imaging modality that provides excellent tissue differentiation in the detection of orbital pathologies. MRI is advantageous in detecting, identifying, and differentiating lesions from normal tissue. In addition, since it does not contain ionizing radiation, it can be applied in pregnant and children and is reproducible [19]. MRI allows for the detailed examination of soft tissues with high-resolution images, and is especially important for the diagnosis of orbital diseases [20]. Knowing the detailed knowledge of the orbital structures is important for diagnosing orbital diseases. The present study aims to determine the normative orbital structure measurements according to sex in healthy adults using MRI.

## 2. Materials and Methods

The present study included 102 (50 male, 52 female) adult individuals with an average age of 27.38 years (range 18–40) and who applied Adana Yuregir State Hospital for any reason between 2018 and 2019. The measurements were retrospectively performed on T1-weighted images obtained in the axial and coronal planes using MRI. In this study, 204 orbits of 102 normal Anatolian individuals’ MR images were evaluated.

As per article 32 of the Declaration of Helsinki, for medical research using identifiable human material or data, such as research on material or data contained in biobanks or similar repositories, where consent would be impossible or impracticable, the research may be performed only after consideration and approval of a research ethics committee. Therefore, informed consent was waived by the concerned ethics committee. Repository images were used in this retrospective study. Necessary permission was obtained from the Cukurova University Non-Interventional Clinical Research Ethics Committee (2019, No. 86). This study was conducted in accordance with the principles of the Declaration of Helsinki. All methods were performed in accordance with the relevant guidelines and regulations.

Individuals who had any disease associated with the orbit, thought to affect the orbit (e.g., thyroid disease), or who had any pathologies diagnosed by the radiologist that would affect the measurements were excluded from the study. Additionally, among the repository archive images, the experienced radiologist excluded images with a head position that would affect the measurements. Measurements were performed at the same magnification setting in all images by an experienced radiologist (S.T.). All measurements were performed twice by a radiologist with >20 years of experience, and the averages were recorded (S.T.). The contours of the muscles were also determined by an experienced radiologist (S.T). However, since the superior rectus and levator palpebrae superioris muscles could not be distinguished precisely, they were measured together as a single muscle group, named the superior muscle group.

A 1.5-T MRI System (GE Signa Excite HD; GE Medical Systems, Milwaukee, WI, USA; TE: 20, TR: 200, FOV: 24, slice thickness: 10, acquisition time: phase: 256, frequency: 256) was used for the MRI. It enables the fastest pulse sequence play-out, 8-channel imaging, and widest selection of phased-array coils. It is shadowed to facilitate reading. The axial images were parallel to the optic nerve, whereas the coronal images were perpendicular to the axial images. Images that did not comply with the standards or were not clear enough were selected by a radiologist and excluded from the study.

**Figure 1:**
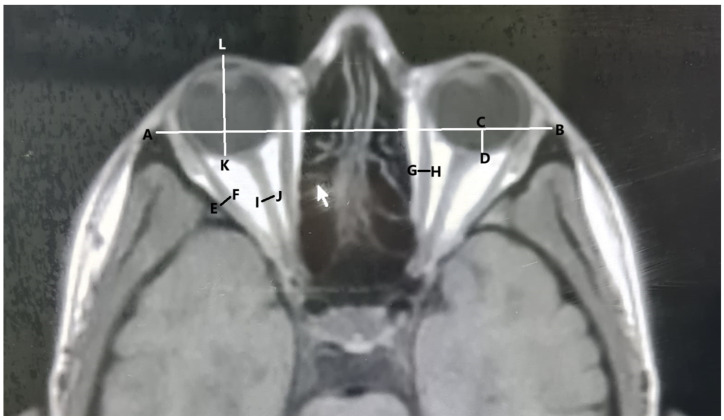
Measurement of orbital structure on axial images (A–B: length of the interzygomatic line; C–D: globe position; E–F: diameter of the lateral rectus; G–H: diameter of the medial rectus; I–J: optic nerve sheath diameter; K–L: ocular bulb length).

**Figure 2:**
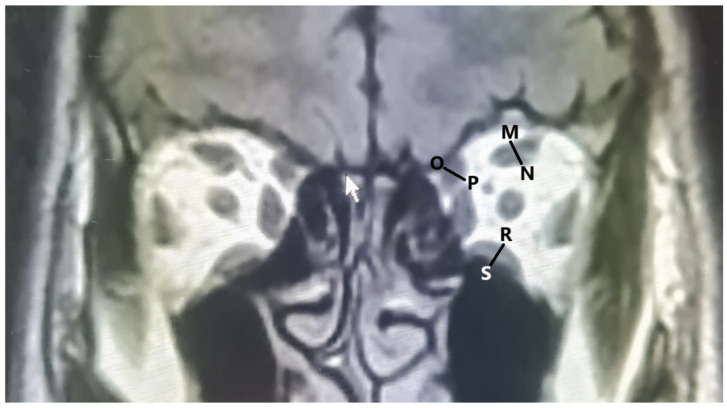
Measurement of orbital structure on coronal images (M–N: diameter of the superior muscle group; O–P: diameter of the superior oblique muscle; R–S: diameter of the inferior rectus).

The following measurements were performed on axial and coronal scans using Karmed PACS (Kardelen Software version 1.62.409, Mersin, Turkey):

- The length of the interzygomatic line: This line was measured on axial images at the mid-globe position (Figure 1).
- Globe position: The distance between the interzygomatic line and posterior margin of the globe. It was measured perpendicular to the interzygomatic line on axial images in the mid-globe position (Figure 1).
- Ocular bulb length: The distance between the posterior surface of the cornea and posterior pole of the ocular bulb was measured in the mid-globe position on axial images (Figure 1).
- Optic nerve sheath diameter: Optic nerve sheath diameter was measured in axial sections from the area where the middle part of the nerve was visible, cutting the nerve course perpendicularly (Figure 1).
- The diameters of the lateral and medial rectus muscles: The horizontal diameter was measured from the widest area in the axial scans (Figure 1).
- The diameter of the superior muscle group: The superior rectus and levator palpebrae superioris muscles were considered together as the superior muscle group, and their measurements were made in this way. Measurements were taken at the widest point in the coronal section, where the muscles were observed together, before entering the fat plane between the two muscles (Figure 2).
- The diameter of the superior oblique: This was measured at the widest point in the coronal section (Figure 2).
- The diameter of the inferior rectus: Measurements were taken at the widest point in the coronal section (Figure 2).

The eye muscle is best visualized when the plane of the section and the course of the muscle are parallel. The courses of the medial and lateral rectus muscles are parallel to the axial plane. Therefore, the medial and lateral rectus muscles were measured on the axial plane. The inferior and superior rectus muscles are not parallel to the sagittal plane. They are visualized best on coronal scans close to their true cross-sections because of the slant angulation of the superior and inferior rectus. In conclusion, we used axial sections for the medial and lateral rectus and coronal sections for the superior group and inferior rectus based on the study of Ozgen and Aydıngoz [21].

The measurements were performed on archived magnetic resonance images. Only axial and coronal magnetic resonance images are available in the archive. Unlike the other extraocular muscles, the inferior oblique muscle runs posterolaterally. However, the oblique images required for the measurement of inferior oblique muscles are not available in the archive. In other words, the available images are not suitable for the measurement of this muscle with posterolateral course. So, it could not be measured [22].

The Statistical Package for Social Sciences (SPSS) IBM 20.0 Chicago, IL, USA was used for the statistical analysis of the study data. The normality of the data distribution was examined using the Kolmogorov–Smirnov test. The Mann–Whitney U test was used to compare data that were not normally distributed according to binary groups, and the independent sample *t*-test was used to compare data that were normally distributed. The paired sample *t*-test was used to compare two dependent data points that had normal distributions, and the Wilcoxon test was used to compare two dependent data points that did not have normal distributions. The relationships between non-normally distributed continuous variables were evaluated using Spearman’s correlation coefficient. The results of the analyses are presented as mean ± standard deviation and median (minimum–maximum) for quantitative variables. The significance level was set at *p* < 0.05.

## 3. Results

The mean values and standard deviations of the diameters of the extraocular muscles, interzygomatic lines, and globe position lengths are listed in Table 1. No statistically significant differences were detected between the other variables, except for the superior oblique (*p* = 0.018).

The mean age of the males was 26.90 years and the mean age of the females was 27.85 years, and there were no statistically significant differences (*p* = 0.541). A statistically significant difference was detected between the males and females in all measurements. All measurements were greater in males than in females. The distributions and comparisons of measurements according to sex are presented in Table 2.

When the relationships between age and orbital parameters were analyzed, small, statistically significant, and positive correlations were detected between age and lateral rectus (r = 0.225; *p* = 0.001) and superior oblique (r = 0.162; *p* = 0.021) muscle strength. No consistent relationships were detected between age and globe position, axial length, interzygomatic line, medial rectus, superior group, inferior rectus, or optic nerve sheath complex (Table 3).

No statistically significant difference was detected between the right and left sides of the measurements (*p* > 0.050). The comparisons of right- and left-side measurements are presented in Table 4.

## 4. Discussion

In this study, the normative values of the orbital structures were determined using T1-weighted MRI. Knowing the dimensions of the orbital structures is necessary for pre- and post-operative reconstruction [8]. The mean values of orbital structures varied according to age, sex, and ethnicity. It is important to know the normal values specific to population in order to understand the expansion of orbital structures. CT, ultrasound, and MRI are used to visualize orbital structures [15]. Among these methods, MRI is frequently preferred for imaging orbital structures because of its advantages such as not receiving ionizing radiation, obtaining multiplane images, and providing high soft tissue contrast [16].

Extraocular muscle enlargement may arise secondary to inflammatory, neoplastic, infective, or vascular problems [5]. It is necessary to know the normative values of the muscle to determine whether there is enlargement of the extraocular muscle [23]. According to the findings of Rana et al. on T1-weighted MR images, the mean length of the medial rectus was 4.1 ± 0.5 mm, the mean length of the lateral rectus was 3.4 ± 0.6 mm, the mean length of the superior muscle group was 4.3 ± 0.7 mm, and the mean length of the inferior rectus was 4.6 ± 0.7 mm. In the same study, they also reported that orbital structures were affected by age and sex, and that the muscle diameters of males were greater than those of females. A positive correlation was detected between age and the lateral rectus, and a negative correlation was detected between age and the medial rectus [5]. The medial rectus was 3.6 ± 0.4 mm, the inferior rectus was 3.7 ± 0.8 mm, the lateral rectus was 2.3 ± 0.5 mm, and the superior oblique was 2.4 ± 0.6 mm according to measurements made by Shen et al. on MRI images in China [15]. In the study, which was performed in Hong Kong, Ko et al. found medial rectus as 3.5 ± 1.3 mm, lateral rectus as 3.2 ± 1.3 mm, superior muscle group as 3.4 ± 1.3 mm, and inferior rectus as 3.8 ±1.7 mm [24]. In another study carried out using CT in the Indian population, the inferior rectus muscles were 3.46 ± 0.64 mm and 3.36 ± 0.62 mm, the lateral rectus muscles were 3.14 ± 0.63 and 3.14 ± 0.73 mm, the medial rectus muscles were 3.8 ± 0.56 and 3.83 ± 0.56 mm, and the superior rectus muscles were 3.78 ± 0.84 and 3.75 ± 0.86 mm (right and left, respectively) [23]. In a study carried out in Korea, Lee et al. reported the medial rectus to be 3.7 ± 1.5 mm, the lateral rectus to be 3.4 ± 1.3 mm, the superior muscle group to be 4.0 ± 1.4 mm, and the inferior rectus to be 4.1 ± 1.6 mm [25]. Rokka et al. found the medial rectus to be 3.72 ± 0.50 mm, lateral rectus as 3.39 ± 0.50 mm, superior muscle group as 3.87 ± 0.38 mm, and inferior rectus as 3.72 ± 0.42 mm in their study among Nepalese subjects [6]. In their MRI study, Ozgen and Aydingoz similarly reported a positive correlation between lateral rectus muscle volume and age, and that the orbital structures of males were significantly larger than those of females. The mean values of the extraocular muscles in the study were as follows: medial rectus, 4 mm; lateral rectus, 3.7 mm; inferior rectus, 4.8 mm; superior muscle group, 4.4 mm; and superior oblique muscle group, 3.2 mm [21]. In their study carried out using CT, Ozgen and Ariyurek reported the medial rectus to be 4.2 mm, the lateral rectus to be 3.3 mm, the superior muscle group to be 4.6 mm, and the inferior rectus to be 4.8 mm [26]. Kızılgöz et al. found the medial rectus as 4.46 ± 0.46 mm, lateral rectus as 4.20 ± 0.51 mm, superior group as 4.67 ± 0.63 mm, inferior rectus as 5.13 ± 0.58 mm, and superior oblique as 2.88 ± 0.44 mm in their MRI based study [7]. The normative MRI data determined in this study are close to the normative values of Ozgen and Aydingoz’s study [21]. Furthermore, our study results were greater than those of the Chinese and Korean population in terms of extraocular muscles [15,25].

The optic nerve sheath diameter was used to evaluate the increase in intracranial pressure noninvasively. The sheath surrounding the optic nerve is a continuation of the subarachnoid space; therefore, changes in intracranial pressure affect the optic nerve sheath complex [14]. In a study conducted by Kim et al. in Korea, the authors reported that the mean optic nerve sheath diameter was 4.71 ± 0.31 mm. They also reported a strong correlation with the eyeball transverse diameter [27]. Additionally, in a comprehensive study that was carried out in Korea using CT images, the average optic nerve sheath complex diameter was found to be 4.2 ± 0.6 mm [25]. Geeraerts reported, in their study carried out in Germany to determine the relationship between optic nerve sheath diameter and intracranial pressure, that the mean optic nerve sheath diameter was 5.08 ± 0.52 mm in healthy adults and that the cutoff value for the presence of intracranial pressure was 5.82 mm [28]. In a study that was carried out in China, the mean optical diameter was reported to be 5.4 ± 0.7 mm [15]. Ko et al. found optic nerve sheath diameter as 4.4 ± 1.7 mm in their CT-based study which was performed in Hong Kong [24]. In a retrospective analysis in India, Gupta et al. reported the mean optic nerve sheath diameter to be 4.72 ± 0.74 mm for the right side and 4.83 ± 0.82 mm for the left side [23]. Rokka et al. found the mean optic nerve sheath diameter as 3.98 ± 0.54 mm in a study performed in Nepal [6]. In another study that was carried out with 200 normal orbits of 100 patients, the mean optic nerve sheath diameter was reported to be 4.4 mm [21]. Ozgen and Ariyurek showed, in their CT-based study, that the diameter of the optic nerve sheath complex was 4.4 mm [26]. Kızılgöz et al. found optic nerve sheath diameter as 5.15 ± 0.58 mm in their MRI based study [7]. In terms of optic nerve sheath complex width, our study results are lower than the results of the studies performed in Germany and China, but similar to the results of the studies performed in Turkey [15,21,28].

Exophthalmos was defined as protrusion of the eyeball forward from the bony orbit [23]. Exophthalmometry is a common method used for the diagnosis and follow-up of thyroid disease. Global positioning is important in the diagnosing of exophthalmometry [29]. The intezygomatic line is used to determine whether hypertelorism occurs as a result of various craniofacial deformities and pathologies (e.g., holoprosencephaly and craniosynostosis) [23]. The mean length of the interzygomatic line was 97.74 ± 3.74 mm in a study that was carried out in Nepal. The distance between the interzygomatic line and the anterior margin was reportedly 16.95 ± 1.48 mm for the right side and 16.86 ± 1.38 mm for the left side. The distance between the interzygomatic line and the posterior margin was 5.68 ± 1.31 mm for the right side and 5.78 ± 1.24 mm for the left side. It was reported, as a result of the study, that the globe position values of the males were significantly greater than those of the females. The same study also reported a statistically significant difference between right- and left-sided measurements [30]. In another study performed in Nepal, the researchers reported that the mean distance between the interzygomatic line and the posterior pole of the globe was 12.8 ± 2.4 mm and the mean interzygomatic line was 94.3 ± 6.1 mm [6]. Gupta et al. reported the mean length of the interzygomatic line to be 96.54 ± 4.40 mm and the distances between the interzygomatic line and posterior margin to be 7.77 ± 2.23 mm on the right and 7.88 ± 2.15 mm on the left [23]. In another study conducted in Korea, the average length of the interzygomatic line was reported to be 105 ± 5.1 mm [25]. Ko et al. reported that the mean distance between the interzygomatic line and the posterior pole of the globe was 9.1 ± 5.0 mm and the mean interzygomatic line was 97.7 ± 7.8 mm in their study, which was conducted in Hong Kong [24]. Ozgen and Aydıngoz reported that the mean distance between the interzygomatic line and the posterior pole of the globe was 8.9 mm, and the average value of the interzygomatic line was 99 mm [21]. In another study carried out on CT images, the interzygomatic line was reported to be 99 mm and the distance between the interzygomatic line and the posterior margin of the globe was 9.4 mm [26]. Kızılgöz et al. found that the mean distance between the interzygomatic line and the posterior pole of the globe was 8.66 ± 2.03 mm and the mean interzygomatic line was 97.96 ± 4.07 mm on their study [7]. In our study, globe position length was found to be greater than the study conducted among a Nepalese population, similar to the study conducted in India. In terms of interzygomatic line length, our study results are greater than the results of the studies performed in India and Nepal and lower than the results of the studies performed in Korea. Additionally, our study results are close to Özgen and Aydıngoz’s study in terms of interzygomatic line length [21,23,25,26].

A limitation of this study is that it was a retrospective study in a limited age group of healthy individuals. With a data review of longer years, it may be possible to determine the normative values of a wider range of age groups and to compare them by including various patient groups. This study measured the medial rectus and lateral rectus on axial images based on the literature. However, there is a need for studies that make measurements in coronal sections, which are considered to be more precise. This is another limitation of this study.

## 5. Conclusions

Although there are many studies in the literature on extraocular muscles, optic nerve sheath complex, and globe position, there are limited studies in the Anatolian population to guide diagnosis and surgical interventions. We believe that a practical and quantitative method can be used to determine the extent of enlargement in the extraocular muscles and optic nerve sheath and the degree of exophthalmos by determining the normative values of the orbital structures in the present study. We thought that the data obtained in this study will help determine pathological conditions such as thyroid disease, increased intracranial pressure, or hypertelorism occurring as a result of conditions such as holoprosencephaly and craniosynostosis.

## Figures and Tables

**Table 1:** Normative orbital measurements for all patients in MRI.

Measurements (mm)	Mean	SD	Median (Min–Max)
Interzygomatic line	99.76	4.63	99.6 (87.90–109.5)
Ocular bulb length	23.08	1.24	23.05 (19.8–26)
Globe position	7.48	1.37	7.20 (5–11.8)
Lateral rectus	3.31	0.57	3.2 (2.3–4.9)
Medial rectus	3.77	0.56	3.7 (2.6–5.2)
Superior group	4.22	0.64	4.2 (2.8–5.8)
Inferior rectus	4.27	0.54	4.2 (3.4–5.8)
Superior oblique	3.56	0.48	3.6 (2.6–4.8)
Optic nerve sheath complex	4.46	0.59	4.5 (3.2–5.6)

**Table 2:** Normative orbital measurements according to sex in MRI.

	Mean ± Standard Deviation (mm)		
Measurement	Male	Female	*p*
Interzygomatic line	102.68 ± 3.89	96.95 ± 3.4	<0.001
Ocular bulb length	23.33 ± 1.32	22.83 ± 1.1	0.005
Globe position	7.66 ± 1.33	7.3 ± 1.39	0.033
Lateral rectus	3.47 ± 0.61	3.15 ± 0.48	<0.001
Medial rectus	3.96 ± 0.52	3.58 ± 0.53	<0.001
Superior group	4.44 ± 0.64	4.01 ± 0.56	<0.001
Inferior rectus	4.47 ± 0.53	4.07 ± 0.48	<0.001
Superior oblique	3.68 ± 0.49	3.45 ± 0.44	0.001
Optic nerve sheath complex	4.65 ± 0.62	4.28 ± 0.51	<0.001

**Table 3:** The relationship between age and orbital parameters.

Measurement	Age	
	r	*p* *
Ocular bulb length	−0.111	0.115
Globe Position	−0.093	0.188
Lateral Rectus	0.225	0.001
Medial rectus	0.13	0.063
Superior Group	−0.014	0.848
Inferior Rectus	0.054	0.447
Superior Oblique	0.162	0.021
Optic nerve sheath complex	0.075	0.285

* Spearman’s Rho correlation coefficient.

**Table 4:** Comparison of right- and left-side measurements.

Measurement	Mean ± SD	Median (Min–Max)	*p*
Globe position (R)	7.49 ± 1.35	7.2 (5–11.7)	0.166 **
Globe position (L)	7.46 ± 1.39	7.2 (5.1–11.8)	
Ocular bulb length (R)	23.08 ± 1.23	23.05 (19.8–25.7)	0.700 *
Ocular bulb length (L)	23.07 ± 1.25	23.05 (20.7–26)	
Lateral rectus (R)	3.28 ± 0.58	3.15 (2.3–4.9)	0.012 **
Lateral rectus (L)	3.33 ± 0.55	3.2 (2.4–4.9)	
Medial rectus (R)	3.77 ± 0.55	3.7 (2.8–5)	0.640 **
Medial rectus (L)	3.76 ± 0.57	3.7 (2.6–5.2)	
Superior group (R)	4.18 ± 0.64	4.2 (2.8–5.8)	0.002 **
Superior group (L)	4.26 ± 0.63	4.3 (2.8–5.8)	
Inferior rectus (R)	4.26 ± 0.55	4.2 (3.4–5.8)	0.196 **
Inferior rectus (L)	4.28 ± 0.54	4.25 (3.4–5.8)	
Superior oblique (R)	3.55 ± 0.47	3.55 (2.6–4.5)	0.018 **
superior oblique (L)	3.58 ± 0.48	3.6 (2.6–4.8)	
Optic nerve sheath complex (R)	4.46 ± 0.61	4.55 (3.3–5.6)	0.962 **
Optic nerve sheath complex (L)	4.46 ± 0.58	4.5 (3.2–5.5)	

* The paired sample *t*-test, ** Wilcoxon test.

## Data Availability

Data are contained within the article.
